# Responses of active soil microorganisms facing to a soil biostimulant input compared to plant legacy effects

**DOI:** 10.1038/s41598-020-70695-7

**Published:** 2020-08-13

**Authors:** Eve Hellequin, Cécile Monard, Marion Chorin, Nathalie Le bris, Virginie Daburon, Olivier Klarzynski, Françoise Binet

**Affiliations:** 1 University of Rennes, CNRS, ECOBIO (Ecosystèmes, Biodiversité, Évolution)-UMR 6553, 35000 Rennes, France; 2 BIO3G Company, 7 rue du Bourg-Neuf, 22230 Merdrignac, France; 3 Present Address: University of Sorbonne, CNRS, EPHE, PSL, UMR METIS, 75005 Paris, France

**Keywords:** Applied microbiology, Bacteria, Environmental microbiology, Fungi, Microbiology, Molecular biology

## Abstract

Agriculture is changing to rely on agroecological practices that take into account biodiversity, and the ecological processes occurring in soils. The use of agricultural biostimulants has emerged as a valid alternative to chemicals to indirectly sustain plant growth and productivity. Certain BS have been shown to select and stimulate plant beneficial soil microorganisms. However, there is a lack of knowledge on the effects and way of action of the biostimulants operating on soil functioning as well as on the extent and dynamic of these effects. In this study we aimed to decipher the way of action of a seaweed and amino-acids based biostimulant intended to be applied on soil crop residues to increase their microbial mineralization and the further release of nutrients. By setting-up a two-phase experiment (soil plant-growing and soil incubation), our objectives were to (1) determine the effects of the soil biostimulant over time on the active soil bacteria and fungi and the consequences on the organic carbon mineralization in bare soils, and (2) assess the biostimulant effects on soil microorganisms relatively to plant legacy effects in planted soils. We demonstrated that the soil biostimulant had a delayed effect on the active soil microorganisms and activated both plant growth promoting bacteria and saprophytes microorganisms at the medium-term of 49 days. However, the changes in the abundances of active microbial decomposers were not associated to a higher mineralization rate of organic carbon derived from soil and/or litter. The present study assessed the biostimulant beneficial effect on active soil microbial communities as similar as or even higher than the legacy effects of either *A. thaliana* or *T. aestivum* plants. We specifically showed that the biostimulant increased the active fungal richness to a higher extent than observed in soils that previously grew the two plants tested.

## Introduction

To cope with the over use of chemical fertilizers that harm ecosystem health and soil functioning, agriculture is more than ever changing to rely on agroecological practices. Agroecology is based on the conservation of biodiversity, the strengthening of biological processes and the looping of biogeochemical cycles^1^. In soil, biogeochemical cycles are partly regulated by saprophyte microorganisms that have an essential role in the decomposition and mineralization of soil organic matter releasing nutrients to plants^2,3^. Fitting with the agroecological principles is the use of biostimulants (BS) intended to select and stimulate beneficial soil microorganisms to indirectly sustain plant growth and productivity^5,6^. According to Yakhin et al.^7^ and Du Jardin^8^, biostimulants are defined as products of biological origin whose function, when applied to plants or soil, is to stimulate natural ecological processes in order to improve nutrient absorption and tolerance to abiotic stress in plants, and to increase the nutritional quality of plants, regardless of the nutrient content of the biostimulant. The literature reports several positive effects of either soil biostimulants or plant biostimulants on plant quality and growth^7,9^. However, the modes of action of biostimulants remain largely unknown partly due to the great diversity of raw materials used in the manufacture of each product^10^. Depending on their raw source material, several categories of biostimulants are described: Humic and fulvic acids, protein hydrolysates of animal or plant origin, N-containing compounds or amino acids, seaweed extracts, plants (seeds, leaves, roots, root exudates) or fruits, chitin and chitosan, microbial inoculants^8,11^.

Biostimulants dedicated to soil are directly applied on crop residues to improve litter decay and further mineralization by soil saprophyte microorganisms, thereby preserving and even increasing the fertility of soil. The efficiency of these biostimulants in agroecology relies on sustainable agricultural practices such as crop returning to the soil to improve contents of soil nutrients and organic matter^12,13^. Unlike fertilizers that directly fuel the plants, soil biostimulants aim to stimulate natural soil processes such as those mediated by microorganisms^14^. Up to now, the mechanisms enrolled to improve soil biological functioning are still misunderstood. In the literature, most of the studies focusing on the impact of soil biostimulant on soil biochemical properties are mainly limited to the analysis of soil enzyme activities as a proxy of microbial functions^5,15,16^. Soil microbial communities are strongly influenced by soil properties and respond, for example, to changes in soil pH or moisture^17,18^. Soil biostimulants can thus act indirectly on soil microorganisms by inducing changes in the physico-chemical properties of soil depending on their composition without having a fertilizing effect. It has been recently reported that the carrageenans contained in algae, are hydrophilic colloids that absorb soil moisture, further sustaining microbial activities^19^.

The biostimulant effects could be then compared to natural regulators of microbial communities as plants that induced legacy effects on soil after their disappearance or harvesting^20^. Plants drive and shape the soil microorganisms by secreting root exudates that either change soil properties, act as signal molecules or can be utilized as nutrient resources by microbes^21^. Because soil properties are plastic, modifications induced by plant may persist after ceasing these direct biotic interactions (i.e. legacy effect)^22^. Thus, as proposed by Philippot et al.^23^, in this study, we went “back to the roots” to observe and learn from natural plant-soil systems and assessed the effects of a biostimulant on soil microorganisms in comparison with the legacy effects in the rhizosphere of two plants phylogenetically distant (*Arabidopsis thaliana* and *Triticum aestivum)*.

Up to date, the few studies that analyzed the effect of biostimulant on soil microorganisms targeted the total microbial community while the subtle effect induced by the biostimulant might be detected on their active part^24,25^. One can hypothesize that the subtle effects induced by biostimulants should rather be observed on active microbial communities that are directly linked to the soil processes than on total communities. In the present study by using RNA-based analyses (RNA metabarcoding and RT-qPCR), we specifically studied the response of active soil bacteria and fungi face to the use of a soil biostimulant, derived from seaweed extracts and intended to improve the mineralization of crops residues returning to soil. We thus performed a two-phase experiment, the soil was first planted or not with *A. thaliana* or *T. aestivum* for one generation, sampled in the vicinity of their roots and secondly tested for straw litter mineralization in soil microcosms. Our objectives were to (1) determine the effects of the soil biostimulant on the active soil microbial communities in a bare soil and the consequences over time on the organic carbon mineralization, and (2) assess these effects comparatively to the plant legacy effects on soil microorganisms. We first hypothesized that not by fueling the microorganisms but by modifying their microhabitats, the biostimulant rapidly affects the abundance and composition of soil active microbial communities leading to an increase in the C mineralization function. Moreover, similarly to the persistent plants legacy effects that affects soil microbial activity and abundance, we expected the biostimulant to act on soil microorganisms in the same order of magnitude.

We thus demonstrated that the biostimulant had a delayed beneficial effect on active soil microorganisms at the medium-term of 49 days. It specifically activated saprophytes bacteria and fungi and bacteria known to promote plant growth. After 49 days, the richness of active fungi was even higher in soil treated with the biostimulant than in the soil that had grew plants. However, these changes did not induce an increase in the mineralization rate of the soil organic carbon.

## Materials and methods

### Soil and biostimulant characteristics

The soil was an agricultural topsoil (0–20 cm depth) collected in April 2018 in the agronomic experimental site of the regional chamber of agriculture of Brittany, at Kerbernez (Plomelin, France) (47° 56′ 39.3″ N/4° 07′ 47.9″ O). After collection, the soil was sieved (mesh size 4 mm), air dried and stored in the dark at 4 °C before being used. Its physical, chemical and biological characteristics were determined according to Carter and Gregorich^26^ and are summarized in Table 1. The biostimulant tested in the present study derived from seaweed extracts and animal amino-acids mixed together with water to give a product with low fertilizing properties. It has been provided for free by the company BIO3G. The biostimulant is in liquid form, composed of natural raw materials without any additives; it is intended for application on crop residues before they are buried in the soil. It was characterized by 29% of dry extract, had a pH_water_ of 6.4 and it contained 25% of organic matter (i.e. 14.5% organic carbon) and 2% of total nitrogen. The biomolecules such as amino-acids and polysaccharides and free sugars composing the biostimulant were determined and provided in Table S1. The polysaccharides were determined by gas chromatography after methanolysis. The polysaccharides contents is high as its represented 40.2% of dry extract, and their methanolysis indicated that they are mainly composed of: glucose, galactose and some mannose as monomers. In addition, the biostimulant also contained several amino acids that represented 9% of dry extract out of which Leucine, Aspartic acid, Lysine, Valine and Alanine. More information on the composition of the BS is given in Table 1.

**Table 1:** Physico-chemical, biochemical and biological properties of the initial soil used for plant growing and incubations and the analytical composition of the biostimulant under study.

	Kerbernez soil	Kerbernez soil	Biostimulant
	Before planted soil phase	After planted soil phase	
**Physico-chemical properties**			
% Clay	12.9	n.d.	n.d.
% Silt	40.3	n.d.	n.d.
% Sand	46.8	n.d.	n.d.
% Organic matter	4.9	n.d.	25
% Dry extract	n.d.	n.d.	29
Tot orgC (mg g dw^−1^)	33 ± 1	32 ± 3	145
Tot N (mg g dw^−1^)	3 ± 0.12	3 ± 0.28	20
Diss tot N (µg g dw^−1)^	20 ± 23	26 ± 0.7	n.d.
Diss NO_3_^−^ (µg g dw^−1^)	5 ± 2	9.5 ± 0.6	n.d.
Diss NH_4_^+^ (µg g dw^−1^)	4 ± 0.2	3.5 ± 0.3	n.d.
Diss orgC (µg g dw^−1^)	85 ± 4	92 ± 2	n.d.
Olsen PO_4_^3−^ (mg g dw^−1^)	69 ± 6	75 ± 1.3	n.d.
P_2_O_5_ (mg kg dw^−1^)	212	n.d.	2000
Ca (mg kg dw^−1^)	1,159	n.d.	1,300
Mg (mg kg dw^−1^)	182	n.d.	400
K (mg kg dw^−1^)	392	n.d.	8,000
S (mg kg dw^−1^)	n.d.	n.d.	2000
B (mg kg dw^−1^)	n.d.	n.d.	5.9
Zn (mg kg dw^−1^)	n.d.	n.d.	13.1
pH_water_	5.5	n.d.	6.4
CEC (meq 100 g^−1^)	10.8	n.d.	n.d.
**Biochemical properties**			
Amino acids (g 100 g dw^−1^)	n.d.	n.d.	9.3
Polysaccharides (g 100 g dw^−1^)	n.d.	n.d.	40.2 ± 2.5
**Microbiological properties**			
MBC (µg g dw^−1^)	221 ± 13	228 ± 3.4	n.d.
MBN (µg g dw^−1^)	17 ± 2	24 ± 1	n.d.
Total ITS (copy g dw^−1^)	2.29E + 07 ± 1.15E + 07	3.92E + 07 ± 2.46E + 06	≤ Detection threshold
Total 16S (copy g dw^−1^)	1.12E + 09 ± 5E + 08	1.72E + 09 ± 2.5E + 08	5.79E + 07 ± 2E + 07
DNA (ng g dw^−1^)	21,323 ± 8,299	21,364 ± 1,850	2069 ± 143
Active ITS (copy g dw^−1^)	9.80E + 07 ± 7.81E + 06	8.86E + 06 ± 1.07E + 06	≤ Detection threshold
Active 16S (copy g dw^−1^)	1.66E + 12 ± 3.84E + 10	5.27E + 11 ± 7.87E + 10	3.36E + 09 ± 1.66E + 09
RNA (ng g dw^−1^)	13,371 ± 1,153	8,208 ± 645	557 ± 40

Means and standard errors, n = 4.

*dw* dry weight, *meq* milliequivalent, *n.d.* not determined.

### Planted soil phase

The soil was first planted by either *A. thaliana* or *T. aestivum* or not planted as the bare soil. The *A. thaliana* seeds from the Ecotype Columbia-O and the *T. aestivum* L. cv. “Baldus” seeds were kindly provided by IsoLife BV (Wageningen, the Netherlands). The *A. thaliana* seeds were surface-sterilized for 7 min in 2.63% sodium hypochlorite solution containing 50% of ethanol 90% and washed twice in ethanol 90%. The *T. aestivum* seeds were surface-sterilized for 1 min in ethanol 90%, 20 min in 1.75% sodium hypochlorite solution containing 0.05% Tween 20 (Sigma-Aldrich) and washed six times in sterile water. The soil was adjusted to 60% of its water holding capacity (WHC), sieved (mesh size 2 mm) and the equivalent of 10 kg of dry soil were used to fill sterile planters. Three planters were used, one for each plant and one for unplanted bare soil. After sowing the seeds directly on soil, these three soils planters were placed in an environmental growth chamber (Percival scientific, Perry, Iowa) for 2 months (63 days) with the following conditions: 16 h light (day), 8 h dark (night) and light intensity of 160 µmol m^−2^ s^−1^ , 20 °C and at a relative humidity of 80%. Soils were watered with sterile water periodically to maintain WHC around 60% by weighting the planters. After 63 days of growing plant, the bulk soil from the bare soil and the soil at the vicinity of the roots of *A. thaliana* and *T. aestivum* were used for setting-up the soil microcosms dedicated to the litter straw mineralization. Four replicates of the initial raw material (soil and biostimulant) and four soil replicates (30 g of dry soil for each replicate) of the bare and planted soils were sampled and stored at − 20 °C and − 80 °C for further chemical and microbial analyses, respectively.

### Soil microcosm incubation phase

The soil microcosm incubations to study the straw mineralization consisted in the equivalent of 30 g of dry soil incubated for 49 days in the dark at 28 °C. A 3 × 2 (without and with straw) factorial design was performed in 4 replicates and repeated for five incubation times (initial soil and 0, 3, 7, 21 and 49 days after the straw and/or biostimulant addition to soil). It corresponded to a total of 240 soil microcosms. The seeds of *T. aestivum* used for plant growing (phase 1) and the straw used for the mineralization kinetics both came from the same variety and batch. The straw was chopped into pieces of 1 mm. To avoid any soil contaminations by external microorganisms, the straw was sterilized by Ionisos (Dagneux, France) with gamma irradiation at 25 KGy. The sterilization efficiency was checked by none amplification of the 16S rRNA gene with a polymerase chain reaction (PCR). Each 30 g equivalent dry soil (e.g. bare soil, and previously planted soils with *A. thaliana* or *T. aestivum*), was mixed with 300 µL of sterilized water or 300 µL of diluted BS, corresponding to an input of 2.6 mg of dry product (i.e. 37.85 µg organic carbon, 50 µg total nitrogen and 5.22 µg phosphorus) and/or with 184 mg of *T. aestivum* chopped straw corresponding to an input of 40 mg of organic carbon and 0.3 mg of organic nitrogen. Each soil microcosm was placed in a hermitically closed 1 L glass jar. At each incubation time, except initial time, the CO_2_ produced and accumulated in the headspace was quantified by micro gas chromatography (Agilent, Santa Clara, California, United States) and expressed as mg of C–CO_2_ g^−1^ dry soil. After each measurement, the air of the headspace was entirely renewed and the soil moisture was maintained at 60% WHC by weighting and adding sterilized water if needed. At day 0 and after each CO_2_ measurement (days 3, 7, 21, 49) a time series of microcosms was destroyed and stored at − 20 °C and − 80 °C for further chemical and microbial analyses, respectively.

### Chemical and nutrient analyses

The total organic carbon and nitrogen contents of the straw litter were determined with an elemental analyzer (Elementar, Vario PYRO Cube, Lyon, France). The microbial carbon and nitrogen biomasses (MBC and MBN, respectively) were determined in all the 240 soil samples using the chloroform fumigation and extraction method described by Vance et al.^27^. Briefly, 10 g of fresh soil were fumigated with chloroform gas. The dissolved organic carbon and nitrogen were extracted with a K_2_SO_4_ (0.5 M) solution. The dissolved organic carbon from the soil (non-fumigated) and from the soil microorganisms (fumigated soil) were measured by a Total Organic Carbon analyzer (Bioritech, Voisis-le-Bretonneux, France). For the dissolved total nitrogen, 10 ml of the extracts were oxidized to NO_3_^−^ with 0.2 M K_2_S_2_O_8_, 0.5 M H_3_BO_3_ and 0.4 M NaOH and autoclaved 2 h at 115 °C. The NO_3_^−^ was then measured by an automated photometric analyzer (Gallery plus, Waltham, Massachusetts, USA). The MBC was calculated using the equation of Vance et al.^27^:

1 $$MBC =\left(Cf-Cnf\right)Kc$$

The MBN was calculated using the equation of Brookes et al.^28^:

2 $$MBN=\frac{\left(Nf-Nnf\right)}{Kn}$$

where *Cf* and *Nf* are the dissolved organic carbon and nitrogen in fumigated soil, *Cnf* and *Nnf* are the dissolved organic carbon and nitrogen in non-fumigated soil and *Kc* and *Kn* are the correction factors of 2.64 and 0.54, respectively.

The NO_3_^−^ and NH_4_^+^concentrations in soil were measured by an automated photometric analyzer following K_2_SO_4_ extraction. According to Olsen et al.^29^, the PO_4_^3−^ was extracted from 5 g of fresh soil in 5 mL of NaHCO_3_ (1 N) solution. The pH was then adjusted to 5 with a H_2_SO_4_ (5 N) solution. Distilled water was added to obtain a final volume of 20 mL and 4 mL of a reagent B prepared with 200 mL of a reagent A (12 g [NH_4_]_6_Mo_7_O_24_·4(H_2_O) (5 × 10^–3^ N), 0.29 g C_8_H_10_K_2_O_15_Sb_2_(2 × 10^–4^ N) in 1 L of a H_2_SO_4_ (5 N) and distilled water for a final volume of 2 L) with 1.056 g C_6_H_8_O_6_ (0.03 N) were added. A blue coloration was obtained after 10 min at room temperature and the optical density was measured at 720 nm with a spectrophotometer Uvikon XS Secomam (BioServ, Morangis, France). The PO_4_^3−^ concentration was determined with a standard curve of concentrations values ranging from 2.5 to 12.5 mg PO_4_^3−^ L^−1^.

### DNA and RNA extractions and library construction

The DNA and RNA were co-extracted from 500 mg of − 80 °C frozen soils following the Griffiths et al.^30^ protocol with some modifications according to Nicolaisen et al.^31^ and Monard et al.^32^. Lysing Matrix E tubes (MP Biomedicals, California, USA) were used for the cell lysis and agitated at 30 m s^−1^ for 3 min in a bead beating. Glycogen (0.1 mg) was added to precipitate nucleic acids for 2 h at 4 °C that were subsequently pelleted by centrifugation at 18,000g for 30 min at 4 °C. Nucleic acids were resuspended in 50 µL of DNase–RNase-free water. The biostimulant DNA and RNA were extracted from 3 mL of pure product using a protocol adapted from Quaiser et al.^33^ consisting in the addition of 25 mL of lysis buffer (Cetyltrimethylammonium bromide (CTAB) 4%, Polyvinylpyrrolidone (PVP) 0.5%, NaCl 0.75 M, potassium-phosphate 100 mM (50:50 of K_2_HPO_4_:KH_2_PO_4_), Ethylenediaminetetraacetic acid (EDTA) 20 mM, β-mercaptoethanol 1%, guanidine thiocyanate 1 M) preheated at 65 °C. After 30 min at 65 °C and intermittent vortexing every 5 min, one volume of chloroform-isoamylalcohol (24:1) was added, the samples were mixed by vortexing for 1 min and centrifuged during 30 min at 4,500 rpm. The aqueous phase was recovered and the DNA and RNA were precipitated by adding 0.5 volume of pure ethanol. The extracts were purified using the illustra MicroSpin Column kit (GE Healthcare) according to the manufacturer’s instructions. The DNA and RNA qualities were assessed on a 1% agarose gel and on a nanodrop spectrophotometer (ND-1000, Nyxor Biotech, Palaiseau, France). DNA quantity was assessed by the Qubit fluorimetric (Invitrogen, Carlsbad, California, USA) following the instruction of the broad range quantification. Half of the extract was stored at − 20 °C before further DNA-based analyses on (initial raw material and 0 days samples); the other half was subjected to a DNAse treatment according to the manufacturer’s instructions (Promega, Madison, WI, USA) for further RNA-based analyses on (initial raw material and—0, 3, 7, 21 and 49 days samples). The absence of DNA was checked by none amplification using PCR on the 16S rRNA gene. RNA quantity was assessed by the Qubit fluorimetric and 200 ng of RNA were used to run the reverse transcription (RT) according to the manufacturer’s instructions (RevertAid RT kit, Thermo Scientific, USA). Half of the cDNA products was stored at − 20 °C to prepare 16S and ITS1 rRNA amplicons for Illumina sequencing and the other half was used for the quantification of the total bacteria and fungi by quantitative PCR (qPCR) as described further.

### Library construction

The bacterial and fungal 16S rRNA and ITS1 libraries were constructed using a two-step PCR approach "16S metagenomics sequencing library preparation" protocol given by Illumina. The following primer sets were used for the bacteria and fungi: 341F (5′-CCTACGGGNGGCWGCAG-3′) and 785R (5′-GACTACHVGGGTATCTAATCC-3′)^34^ and ITS1f. (5′-CTTGGTCATTTAGAGGAAGTAA-3′)^35^ and ITS2 (5′-GCTGCGTTCTTCATCGATGC-3′)^36^. Each primer set contained the following overhang adapters: forward overhang (5′-ACACTGACGACATGGTTCTACA-3′) and reverse overhang (5′-TACGGTAGCAGACTTGGTCT-3′).The first PCR was carried out in two replicates in a total volume of 25 μL containing each bacterial or fungal primer (0.2 µM), 12.5 µL 2X *TransTaq* HiFi PCR SuperMix (1X), 0.5 µL T4 gp32 (100 ng/µL), 2 µL DNA or cDNA and ultrapure water to reach the final volume. The amplification conditions were as follows: for bacteria, 3 min at 94 °C, 30 cycles of 30 s at 94 °C, 30 s at 55 °C and 30 s at 72 °C and a final 10 min extension step at 72 °C; for fungi, 4 min at 94 °C, followed by 35 cycles of 30 s at 94 °C, 30 s at 52 °C and 30 S at 72 °C and a final 10 min extension step at 72 °C. The amplicon quality was assessed on a 1% agarose gel and the replicate amplicons were pooled and sent to the Génome Québec Innovation Center for normalization, barcoding, multiplexing and Illumina paired-end (2 × 300 bp) MiSeq sequencing.

### Microbial sequence analysis

The sequences are available on the sequence read archive (SRA) database (Bioproject PRJNA540147). The FROGS pipeline was used to analyze the 512 amplicon sequences of bacteria and fungi^37^. The raw reads were merged with a minimum overlap of 20 bp using FLASH^38^ for bacteria and Vsearch for fungi^39^, filtered according to the following criteria: expected amplicon size of 470 bp for bacteria, minimal length of 400 bp for bacteria and 50 bp for fungi, and maximal length of 580 bp for both bacteria and fungi, respectively. No ambiguous nucleotides were allowed and both the primer sequences and the sequences without the two primers were removed with the Cutadapt tool^40^. The sequences were then dereplicated and clustered using the swarm method^41^ with an aggregation distance equal to 3 for clustering. The chimera were removed using the Vsearch tool with the UCHIME de novo method^42^ combined with a cross-sample validation. The bacterial and fungal OTUs present in at least 4 out of 256 samples and representing at least 0.0005% of all sequences were retained and corresponded to OTUs with a minimum of 16 and 33 sequences for bacteria and fungi, respectively. For fungi, the ITSx tool was used to extract the highly variable ITS subregions from ITS sequences^43^. The taxonomic affiliations were performed with BLAST+^44^ using the 16S SILVA database (Silva 132) for bacteria and the UNITE fungal ITS database release version 7.1 for fungi^45^. At the end of the process a total of 2,395,758 sequences corresponding to 8,914 OTUs and 5,698,502 sequences corresponding to 1,360 OTUs were obtained for bacteria and fungi, respectively.

### Bacterial and fungal quantification

The total bacterial (16S rRNA gene) and fungal (ITS2 region) abundances were quantified by qPCR, runned in duplicate, on the BioRad CFX Connect Real-Time detection System. The qPCR reactions were conducted in a final volume of 15 µL using 1 ng of soil DNA or 2 µL cDNA 10 times diluted (i.e. 0.8 ng RNA) in 1 × iQ SYBR Green Supermix (Bio-Rad Laboratories, USA) with 0.67 mg/mL BSA (New England BioLabs, USA). The following primer sets were used for the bacteria (0.25 µM each) and fungi (0.3 µM, 0.23 µM and 0.075 µM): 341F and 534R (5′-ATTACCGCGGCTGCTGGCA-3′)^46^, gITS7 (5′-GTGARTCATCGARTCTTTG-3′)^47^, ITS4 (5′-TCCTCCGCTTATTGATATGC-3′)^36^ and ITS4a (5′-TCCTCGCCTTATTGATATGC-3′)^47^, respectively. The qPCR program for bacteria consisted in 5 min at 95 °C, followed by 34 cycles of 15 s at 95 °C, 30 s at 60 °C, 30 s at 72 °C, 5 s at 80 °C and a final melting curves step with an increase of 0.5 °C/5 s from 65 °C to 95 °C. For fungi, the qPCR program consisted in 5 min at 95 °C, followed by 35 cycles of 15 s at 95 °C, 30 s at 56 °C, 40 s at 72 °C, 5 s at 78 °C and the final melting curves step. Standard curves for each assay were generated by serial dilutions to obtain numbers of copies ranging from 10^8^ to 10^2^ of linearized plasmids with cloned fragments of the genes of interest (R^2^ = 0.98 and 0.99 for bacteria and fungi, respectively).The quantities of ITS and 16S rRNA gene fragments were expressed in gene copy numbers per g of dry soil and used to replace the normalized number of sequences obtained from the metabarcoding analysis in each samples.

### Data analysis

All the statistical analyses were performed using R (v3.4.3, Core Team, 2017). The number of sequence per sample was randomly subsampled to the lowest number, soils, BS samples and bacterial and fungal DNA and cDNA amplicons being normalized separately using the *rrafey* function from the vegan package version 2.5-4^48^; 6,399, 15,589, 4,846 and 3,727 for bacterial soil DNA, fungal soil DNA, bacterial BS DNA and fungal BS DNA, respectively, and 7,000, 6,000, 9,154 and 2,766 for bacterial cDNA, fungal soil cDNA, bacterial biostimulant DNA and fungal biostimulant DNA, respectively. Thirty-three samples out of the 512 total ones were below these thresholds and were removed for the further analyses. The rarefaction curves at each sampling date and before and after normalization was performed using the *rarecurve* function from the vegan package and are provided in Figures S1 and S2. For each sample, the absolute abundance of each OTU was calculated based on its relative abundance from the sequencing analysis and to the bacterial 16S and fungal ITS quantifications performed simultaneously. For biostimulant samples, the absolute abundances were expressed in number of added copy by the product per gram of dry soil. The Shannon diversity index was used to estimate the alpha diversity and the richness was estimated as the number of OTUs.

To test for statistical differences between soil treatments we used analysis of variance (ANOVA) and post-hoc Tukey tests on the following parameters: C–CO_2_ emissions, bacterial and fungal richness and Shannon diversity, microbial carbon and nitrogen biomasses, total and active bacterial and fungal abundances and nutrient contents (NO_3_^−^, NH_4_^+^, PO_4_^3−^). To compare the effect of the biostimulant in the bare soil to the legacy effects of the plant on the composition of fungal community at day 49, we generated two powered partial least square discriminant analyses (PPLS-DA), one based on soil samples without straw and the other one on soil samples with straw. The individual plots coupled to a statistical permutation test based on a cross-model validation were used to identify significant differences between the effects of BS and those of plants and we identified the OTUs significantly responsible for these differences with ANOVAs carried out on each OTU abundance.

## Results

### Effect of the biostimulant on straw mineralization and soil nutrient contents

**Figure 1:**
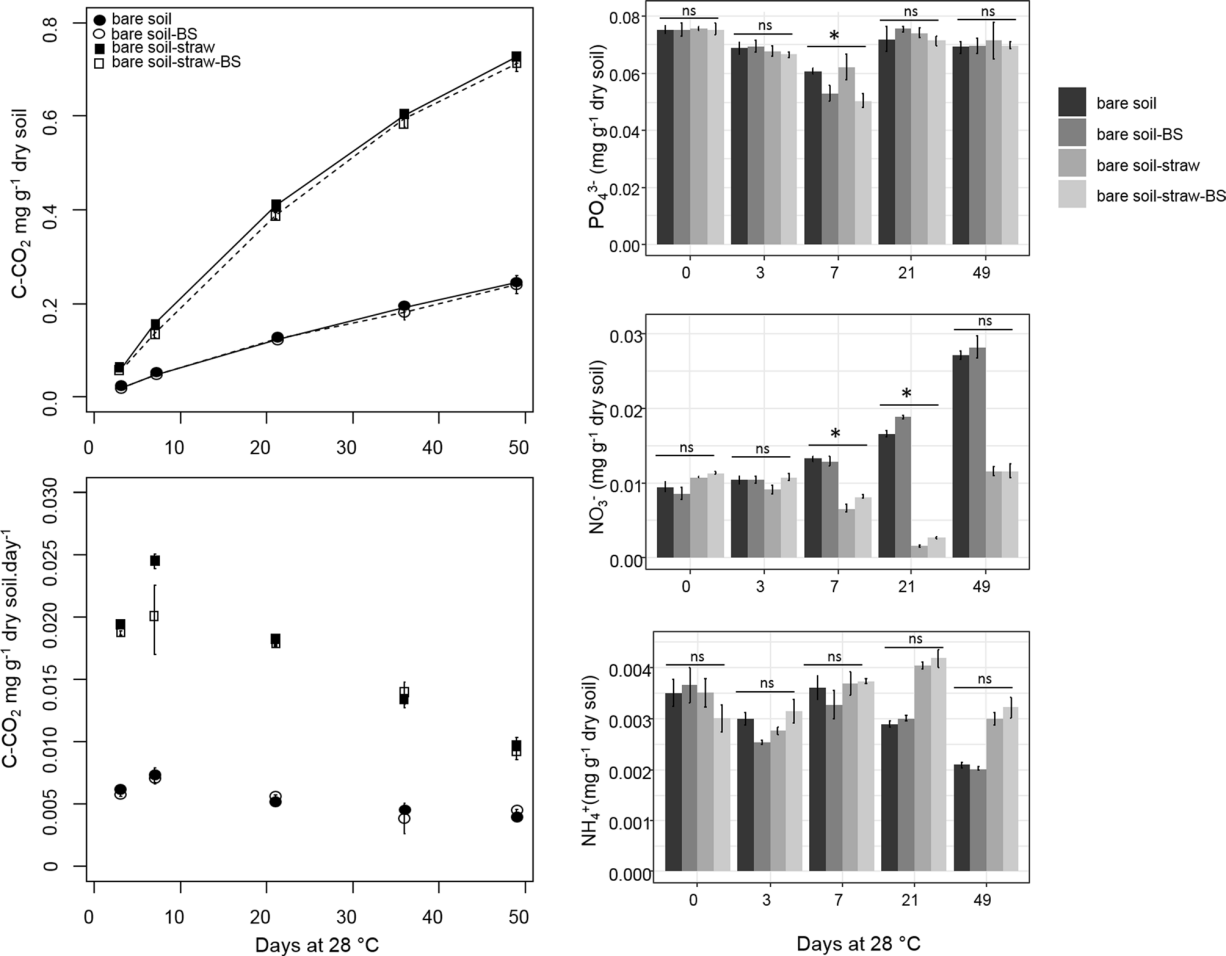
Cumulative kinetics and daily emissions of C–CO_2_ and nutrient contents (N0_3_^−^, NH_4_^+^, PO_4_^3−^) in the bare soils with and without straw or/and biostimulant. The statistical analyses were performed at each sampling date. The error bars indicate the standard errors and the stars indicate significant differences according to ANOVA and Tukey tests, *ns* non-significant. *BS* biostimulant.

The addition of biostimulant (BS) did not affect the mineralization of the soil organic carbon as shown by the cumulative C–CO_2_ emissions after 49 days of incubation at 28 °C: a total of 0.24 ± 0.00, 0.24 ± 0.06, 0.73 ± 0.03 and 0.71 ± 0.05 mg C g^−1^ dry soil was mineralized in the bare soil, bare soil-BS, bare soil-straw and bare soil-straw-BS, respectively (Fig. 1). The daily emissions of C–CO_2_ at the earlier stages (from day 3 to 7) were higher than at the latter stages (from day 21 to 49). A significant effect of the BS was only observed in presence of straw at 7 days, with a lower rate in the daily emission of C–CO_2_ (Fig. 1, ANOVA, F = 190.6, P < 0.001) that matched with a lower content of PO_4_^3−^ and a higher content of NO_3_^−^ (Fig. 1, ANOVA, F = 3.8, *P* < 0.05 and F = 54.3, P < 0.001, respectively). Higher NO_3_^−^ contents with the BS were also observed at 21 days of incubation independently of straw addition (Fig. 1, ANOVA, F = 1,089, P < 0.001). No effect of the BS on the content of NH_4_^+^ in soil was observed along the incubation period. NH_4_^+^ contents were tenfold lower than NO_3_^−^ and were not affected by the BS along the incubation (Fig. 1). Looking at the soil nutrients contents over time, additional ANOVAs and Tukey tests showed that all soils exhibited similar time dynamics of PO_4_^3−^ contents but not for the mineral NO_3_^−^ and NH_4_^+^ contents. In all soils, PO_4_^3−^ contents dropped at day 7 before recovering to almost initial contents at day 49 but with marked differences in the depletion intensity between soils with or without BS. At the contrary, time variation of soil NO_3_^−^ and NH_4_^+^ contents depended on straw addition, the NO_3_^−^ increasing during soil incubation without but not with straw (Fig. 1).

### Immediate effect of biostimulant addition on soil microbial communities

**Figure 2:**
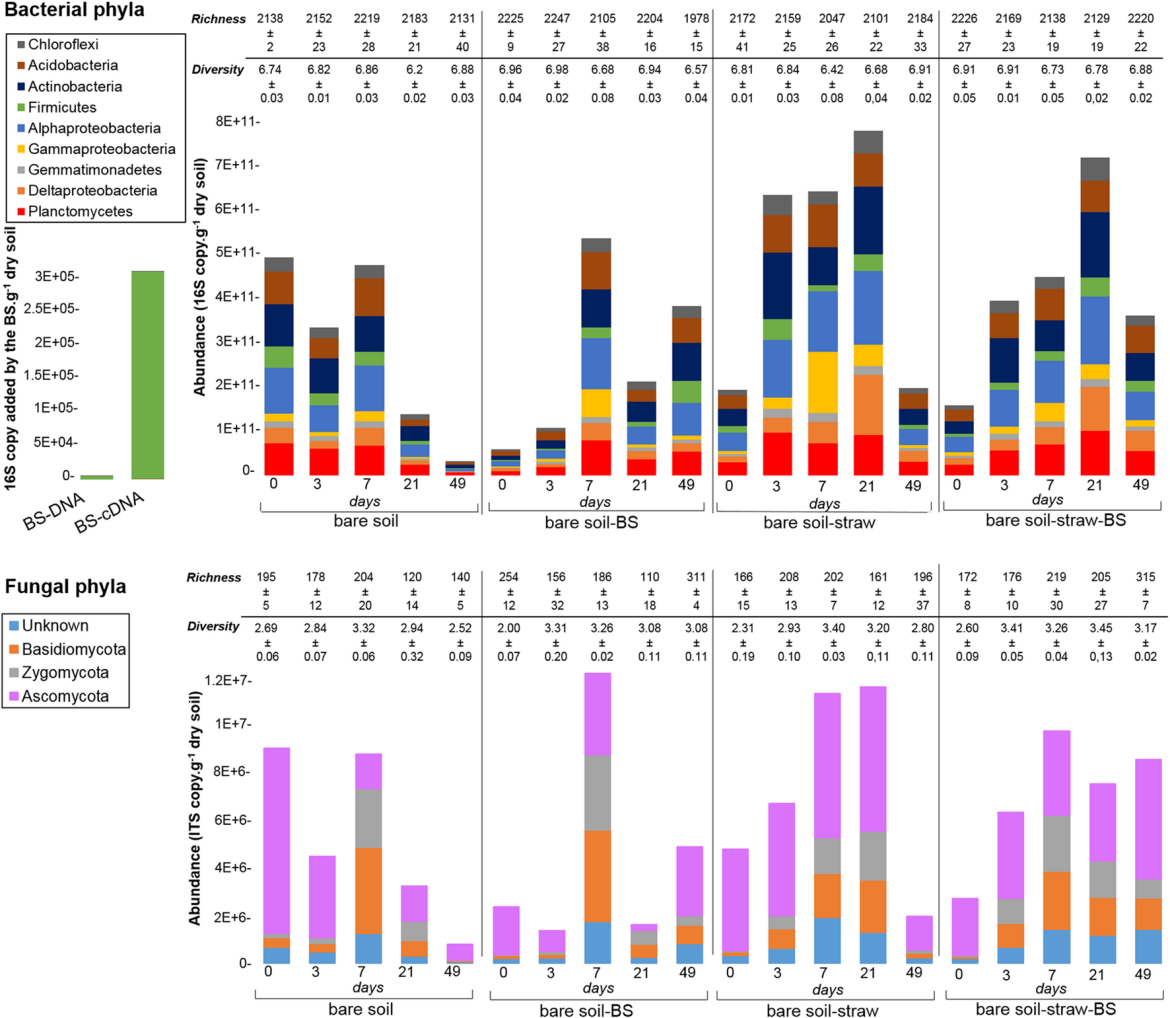
Composition of the active bacterial and fungal communities at the phylum level, richness and Shannon diversity index in the control bare soil with and without straw and/or BS and in the raw BS The abundances were expressed in number of 16S or ITS cDNA copy per gram of dry soil. For the BS, the bacterial abundance was expressed in number of 16S rRNA gene or cDNA copies brought by the BS per gram of dry soil. For fungi, the number of ITS gene and cDNA copies were under the threshold detection of the qPCR thermocycler.

The total and active bacterial communities detected in the BS were both only composed of *Firmicutes* (belonging mainly to *Enterococcaceae*, *Carnobacteriaceae* and *Lactobacillaceae* families); the dose of BS that was applied to the soil represented an input of 2.91 × 10^5^ 16S copy per gram of dry soil of active *Firmicutes* (Fig. 2, left part). The fungal communities in the BS were mainly composed of *Ascomycota* (data not shown), but the fungal abundance in the BS was too low to be detected by the qPCR reaction.

The addition of the BS to the bare soil without straw (0 days), immediately induced a tenfold and twofold decrease in the bacterial 16S and the fungal ITS cDNA copy numbers, respectively (ANOVA, F = 11.4, *P* < 0.001; ANOVA, F = 5.9, *P* = 0.01; Fig. 2). At the meantime, we observed an increase in the active bacterial diversity (from 6.74 ± 0.03 to 6.96 ± 0.04) and in the OTU richness of active bacteria and fungi (from 2,138 ± 2 to 2,225 ± 9 and from 195 ± 5 to 254 ± 12 bacterial and fungal OTUs, respectively; ANOVAs, F = 3.62, *P* = 0.05; F = 12.4, *P* < 0.001; Fig. 2). These changes in active microbial descriptors did not affect the microbial carbon (MBC) and the microbial nitrogen (MBN) biomasses (data not shown). Moreover, the addition of BS to soil did not induce major changes in the phylogenetic composition of the soil microbial community. For example, (1) the *Firmicutes* community was not affected while the BS was only composed of members of this phylum (Figure S4) and (2) the 20 dominant bacterial OTUs present and/or active in the BS were either not detected or at least not stimulated in the soil samples that were treated with BS (Figure S5).

Interestingly, the addition of straw to the soil (Fig. 2, days 0 and 3) lead to similar immediate changes in the active fungi and bacteria communities than those observed with the BS input with the difference that the reduced abundances of active bacteria and fungi and the enhanced bacterial OTU richness were of lower magnitude. Likely to the BS input, no immediate effect of straw addition to the bare soil on MBC and MBN was observed. When combined to the straw addition, the BS effects on active microorganisms were attenuated.

### Dynamics of the active microbial community following biostimulant addition to soil

Along the incubation, bacterial and fungal successions occurred simultaneously. In the bare soil without straw, the total active bacteria and fungi exhibited similar dynamics; their abundances decreased in the first three days of incubation prior peaking at 7 days, and this stimulation was even higher in presence of the BS. After the pivotal time of 7 days the BS significantly sustained higher abundances of both active bacteria and active fungi at the long term (49 days) (ANOVA, F = 8.3, *P* = 0.003) while at the same time they crashed in the bare soil. Those changes in the abundances were associated with significantly lower diversity and richness of active bacterial communities in presence of BS than without (ANOVAs, F = 26.58, P < 0.001; F = 18.2, P < 0.001). Conversely, the BS induced an increase in the diversity and richness of active fungal communities (ANOVAs, F = 15.3, P < 0.001; F = 20.7, P < 0.001, Fig. 2).

In presence of straw, a different dynamic of active microorganisms was observed; the abundances of bacteria and fungi increased until 21 days of incubation and then significantly decreased at 49 days. A delayed positive effect of the BS on the abundances of both active bacteria and fungi was again observed; however these changes in abundances were only significant for the abundance of fungi and associated to an increase of their richness (from 196 ± 37 to 315 ± 7 OTUs without and with BS, respectively (ANOVAs, F = 3.6 , P < 0.05; F = 20.7, P < 0.001, Fig. 2).The combined addition of straw and BS either reduced or faded the bacterial and fungal inhibition observed at 49 days in soil without any input.

The community of soil active fungi was dominated by *Ascomycota*, *Basidiomycota* and *Zygomycota* and the soil active bacterial community was dominated by the *Alphaproteobacteria*, *Acidobacteria*, *Actinobacteria* and *Planctomycetes.* But these taxa had contrasted dynamics over time according to soil treatments (Fig. 2). The BS activation observed at 7 days without straw corresponded to higher abundances of active *Gammaproteobacteria*, in particular members of the *Cellviobrionacaeae* family in presence of BS. Interestingly, in soil that received BS the activity of the *Bacillaceae* (genus *Bacillus*), the dominant family of *Firmicutes* that was detected, increased with time even if they were not initially present in the BS (Figure S4). At the long term (49 days), the relative abundance of active *Ascomycota* (*Sordariomycetes*) was reduced to the benefit of active *Zygomycota* (*Mortierellaceae*) and *Basidiomycota* (*Tremellomycetes*).

### Effects of the biostimulant on soil microorganisms relative to plant legacy effects

**Figure 3:**
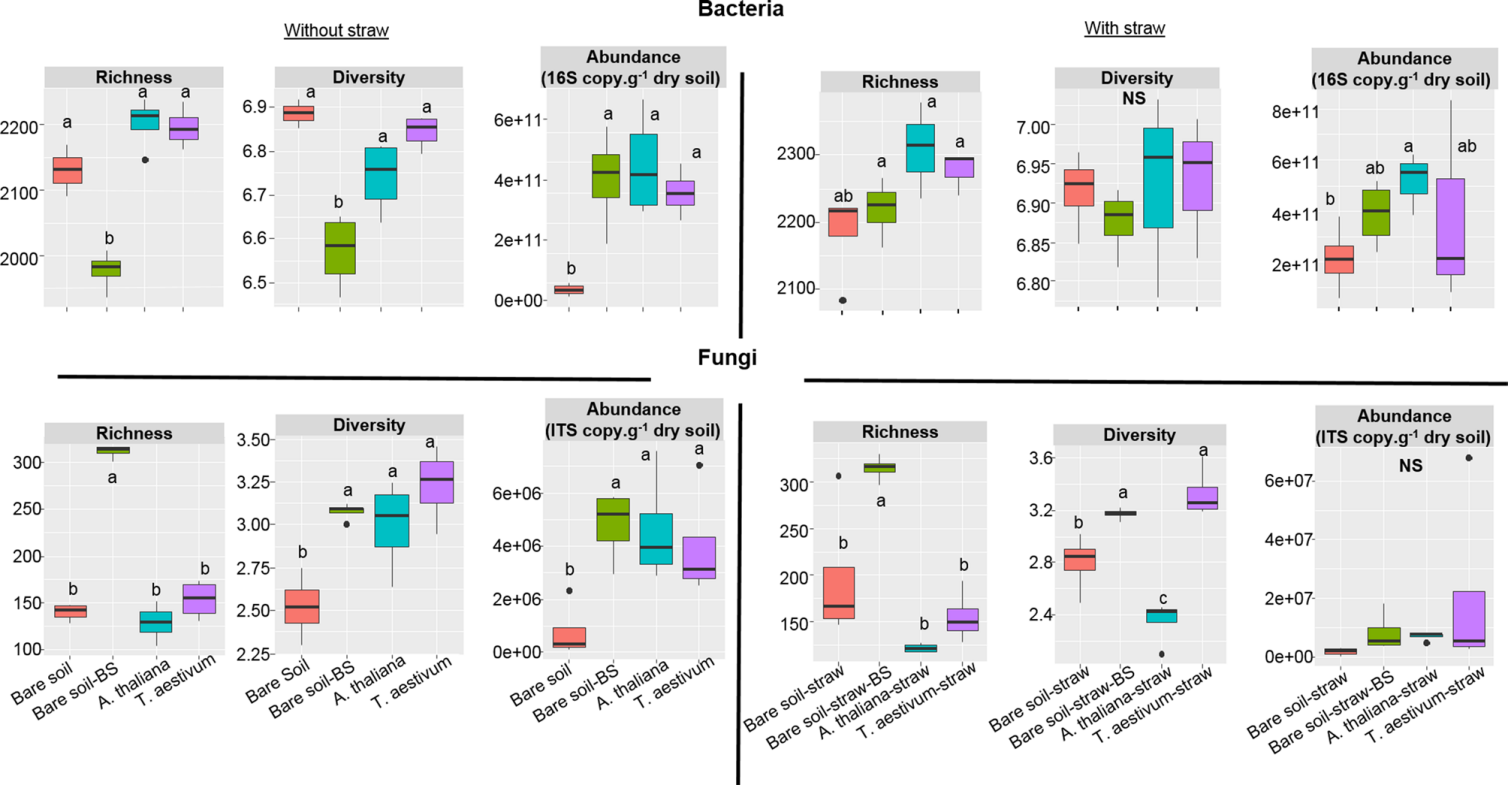
Boxplot of the active bacterial and fungal richness, diversity and abundances in the bare soil with or without BS, and the soils that grew *A. thaliana* or *T. aestivum* without or with straw after 49 days of incubation.

Because we repeatedly observed an activation of both bacteria and fungi at the long term (49 days) with the BS, this incubation time was selected for assessing the BS effects to plant legacy effects. Thus, the effects of the BS and plant legacy on the abundances, diversity indexes and richness of active microorganisms of soils incubated with or without straw were compared (Fig. 3). At 49 days of incubation, we observed a legacy effect for both *A. thaliana* and *T. aestivum* that corresponded to an increase in the abundances of active bacteria and fungi and in the fungal diversity compared to the bare soil (Fig. 3). In soils without straw, both *A. thaliana* and *T. aestivum* had a similar legacy effect, increasing to the same extent the fungal diversity and the abundances of active bacteria and fungi (Fig. 3). This natural changes indirectly induced by the plant was also observed with the BS alone. Depending on the plant that previously grew in the soil and the addition of straw or not, the BS effects on the bacterial descriptors were either lower or to the same extent of those of the two plants (Fig. 3). For example, the abundances of active bacteria in presence of the BS were higher than in the bare soil and were similar to those of soils that previously grew both plants (Fig. 3). By considering soil active fungi, the effect of the BS was sometimes even higher than those of the two plants, it increased fungal richness to a higher extent than observed in soils that grew *A. thaliana* and *T. aestivum* whatever the presence of straw or not. This effect was specific to the BS since, the composition of active fungal communities in these different samples after 49 days of incubation was significantly different (PPLS-DA, *P* = 0.007, 84% of variance explained and PPLS-DA, *P* = 0.006, 91% of variance explained for the treatments without and with straw, respectively; Table 2, Figure S6). The most abundant OTUs that contributed significantly to the differences in the composition of the active fungal communities were mainly detected in the bare soil with BS and affiliated to *Ascomycota* (cluster 1, 24, 25, 29, 30, 97 and 42) without any straw, and to *Ascomycota* (cluster 1, 42, 97, 114, 131, 170), *Basidiomycota* (cluster 46, 126), *Rozellomycota* (cluster 25, 195) and *Zygomycota* (cluster 71) in presence of straw. The other OTUs that contributed to a lower extent to the differences observed were more representative of the soils that grew plants and affiliated to *Zygomycota* (*Mortierellacae*) (cluster 11 and 13) in soil without straw and to *Ascomycota* for *A. thaliana* (cluster 8) and *Zygomycota* for *T. aestivum* (cluster 13) in soil with straw.

**Table 2:** Fungal affiliation at the genus and phylum levels and the abundances expressed in number of ITS cDNA copy per gram of dry soil of the most abundant OTUs that contributed significantly to the differences observed between soils according to ANOVAs.

Cluster number and affiliation	Without straw at 49 days			
	Bare soil	Bare soil-BS	*A. thaliana*	*T. aestivum*
1-Unknown genus of *Sordariomycetes* (*Ascomycota*)	3.6E + 05 ± 2.3E + 05	1.9E + 06 ± 2.9E + 05	1.2E + 04 ± 5.8E + 03	1.3E + 04 ± 5.0E + 03
11-*Mortierella* (*Zygomycota*)	4.4E + 03 ± 2.2E + 03	6.7E + 04 ± 1.1E + 04	2.7E + 05 ± 7.9E + 04	2.2E + 05 ± 4.3E + 04
13-*Mortierella* (*Zygomycota*)	2.6E + 03 ± 2.3E + 03	3.6E + 04 ± 5.2E + 03	4.2E + 05 ± 1.5E + 05	2.7E + 05 ± 6.1E + 04
15: *Sarocladium* (*Ascomycota*)	3.2E + 04 ± 1.2E + 04	1.2E + 05 ± 1.6E + 04	0	0
24: *Epicoccum* (*Ascomycota*)	1.1E + 04 ± 7.6E + 03	3.2E + 04 ± 5.2E + 03	0	3.8E + 03 ± 3.8E + 03
25: Unknown	7.7E + 01 ± 7.7E + 01	1.6E + 05 ± 2.9E + 04	7.3E + 03 ± 7.3E + 03	3.4E + 03 ± 1.4E + 03
29-*Fusarium* (*Ascomycota*)	1.8E + 04 ± 1.4E + 04	5.9E + 04 ± 8.3E + 03	9.2E + 02 ± 9.2E + 02	5.8E + 03 ± 2.9E + 03
30-*Fusarium* (*Ascomycota*)	1.1E + 04 ± 8.5E + 03	3.9E + 04 ± 8.2E + 03	1.9E + 02 ± 1.9E + 02	0
42-*Metarhizium* (*Ascomycota*)	3.6E + 02 ± 2.8E + 02	3.0E + 04 ± 4.6E + 03	1.4E + 04 ± 1.9E + 03	8.4E + 03 ± 5.0E + 03
97-Unknown	3.6E + 01 ± 3.6E + 01	5.3E + 04 ± 7.2E + 03	0	0

	With straw at 49 days			
	Bare soill straw	Bare soil-straw-BS	*A. thaliana*-straw	*T. aestivum*-straw
1-Unknown genus of *Sordariomycetes* (*Ascomycota*)	8.1E + 05 ± 2.5E + 05	3.1E + 06 ± 1.3E + 06	4.8E + 03 ± 3.6E + 03	3.7E + 05 ± 3.6E + 03
8-Unknown genus of *Chaetomicaeae* (*Ascomycota*)	8.2E + 04 ± 2.9E + 04	2.4E + 05 ± 8.0E + 04	3.4E + 06 ± 3.3E + 05	9.9E + 05 ± 2.6E + 05
13-*Mortierella* (*Zygomycota*)	9.6E + 03 ± 5.1E + 03	5.9E + 04 ± 2.2E + 04	2.4E + 05 ± 4.4E + 04	1.1E + 05 ± 2.8E + 04
25: Unknown	3.0E + 04 ± 2.9E + 04	2.9E + 05 ± 1.0E + 05	1.2E + 04 ± 5.8E + 03	2.1E + 02 ± 2.1E + 02
42-*Metarhizium* (*Ascomycota*)	5.9E + 03 ± 4.7E + 03	4.9E + 04 ± 1.4E + 04	4.5E + 03 ± 2.3E + 03	1.1E + 04 ± 7.2E + 03
46-Unknown	2.9E + 03 ± 2.7E + 03	3.1E + 04 ± 1.0E + 04	6.3E + 03 ± 2.2 + 03	7.9E + 03 ± 2.5E + 03
71-*Mortierella* (*Zygomycota*)	5.1E + 03 ± 5.1E + 03	2.3E + 04 ± 6.6E + 03	2.1E + 03 ± 1.4E + 03	0
97-Unknown	1.0E + 04 ± 1.0E + 4	1.1E + 05 ± 3.8E + 04	0	0
114-Unknown	6.7E + 02 ± 6.7E + 02	1.1E + 04 ± 4.6E + 03	0	0
126-Unknown	2.9E + 03 ± 2.9E + 03	4.2E + 04 ± 1.6E + 04	0	1.2E + 03 ± 1.2E + 03
131-Unknown	2.2E + 03 ± 2.2E + 03	4.1E + 04 ± 1.5E + 04	0	0
170-Unknown	4.3E + 03 ± 2.5 E + 03	3.2E + 04 ± 9.5E + 03	0	1.5E + 03 ± 5.3E + 02
195-Unknown	2.2E + 03 ± 2.2E + 03	1.2E + 04 ± 4.5E + 03	0	0

## Discussion

In the present study we tested the effect of a seaweed extracts and amino-acids based soil biostimulant on the dynamics of active soil bacteria and fungi and the consequences on the soil organic carbon and straw mineralization, and the resulting nutrient releases. The biostimulant effects were then compared to the plant legacy effects as natural persistent interactions between plants and soil microorganisms.

### Immediate effect of BS addition on soil microbial communities and straw mineralization

The biostimulant was composed of active *Enterococcaceae*, *Carnobacteriaceae* and *Lactobacillaceae* (Firmicutes). The *Enterococcaceae* family identified in the biostimulant is commonly used during bioprocesses for converting the carbohydrate fraction of lignocellulosic biomass into biofuels^49^. Both *Carnobacteriaceae* and *Lactobacillaceae* are involved in the food fermentation and are constituent of human and animal guts^50^. *Carnobacteriaceae* have also been previously detected in seawater and marine sediment. The biostimulant being partly composed of seaweed extracts it could explained the presence of this bacterial family. These three families probably originated from the raw materials or were introduced during the biostimulant manufacturing process. The microorganisms present in the biostimulant were quantified in very low abundances in soil samples following its addition and whatever the incubation time (10^5^ versus 10^11^). This observation indicates that the biostimulant effect was not primary due to the inoculation of its own microbial communities to soil. It rather induced changes in the native soil microbial communities by activating some specific bacteria and fungi. This is relevant information with respect to the introduction of non-indigenous organisms to soil that requires profound knowledge of the consequences for indigenous soil microorganisms^51^.

The primary significant effect of the BS on the active soil bacteria and fungi was immediate and corresponded to a decline in their abundances that could be explained by its specific physico-chemical composition as it partly sourced from seaweed extracts. Indeed, the high contents of polysaccharides that the biostimulant contained could adsorb temporarily soil microorganisms and therefore explain the immediate decrease of the active microbial abundances^19^. Such an immobilization of microorganisms may provide advantages such as the protection against environmental stress^52^.

Secondly, this immediate effect of the BS on soil microbial community could be due to the physical disturbance induced by the addition of an exogenous material since it was also observed, to a lower extent, after the addition of straw alone. However, any comparison could be intended as we did not find studies investigating the immediate effect of straw addition on soil microorganisms community. Interestingly, in both cases the abundances of active microorganisms immediately decreased but their diversity indexes were maintained or increased suggesting more important impact on dominant microorganisms. Moreover, we demonstrated that the addition of straw to soil reduced or faded the BS effect on the soil microbial community highlighting the importance to study the effect of the BS on soil without straw even if it is intended to be applied on crop residues and litters.

### Dynamic response of the active microbial community following BS addition to soil

Even if we observed a significant decrease in the abundance of active microorganisms following BS input, the soil mineralization function was not immediately affected. All along the incubation time, the soil mineralization kinetics were not increased following the biostimulant addition and the daily emissions of carbon were even lower than observed in the bare soil at 7 days in presence of straw. Still at 7 days, the biostimulant induced changes in the soil stoichiometry with a higher content of NO_3_^−^ and a lower content of PO_4_^3−^ without impacting the soil content of NH_4_^+^. Tian et al.^53^ found that despite variation of C and N content in soils, low soil P content always led to a higher N:P ratio. In the present study, the addition of the biostimulant induced higher N:P ratios and we could have first suggest that the nitrogen that appears in the form of nitrates may come from the mineralization of the biostimulant itself, associated with a consumption of available phosphorus. However, the nutrients that the biostimulant provided to the soil was negligible (e.g. at day 21 the mineral N content increased by 2.34 µg g^−1^ between the control bare soil and control bare soil with BS while the N added by the BS was 1.67 µg g^−1^. At day 7 the P decreased by 7.79 µg g^−1^ between the control bare soil and control bare soil with BS while the P added by the BS was 0.17 µg g^−1^, see Table 1 and Fig. 1). Thus, the higher N:P ratios would rather indicate a P limitation. Indeed, soil microorganisms need enough P to be able to use the organic nitrogen and may also not respond to nitrogen fertilization in soil P-limited^54^. In addition, it has been demonstrated that soil P availability can alter the composition and the diversity of the soil microbial community^55^.

The lack of link between the decrease in the abundance of active microorganisms following BS input and the soil mineralization function could be explained by high diversity and richness of active bacteria and fungi that maintained the mineralization function^56^. This diversity-function relationship is known to vary depending on the function considered and Schimel and Schaeffer^57^ proposed to categorize ecosystem functions as ‘narrow’ (e.g. nitrification) and ’broad ‘(e.g. organic matter decomposition). The organic carbon mineralization is a generalist function supported by a wide range of microbial decomposers^58^. This implies that even if changes in the microbial composition are observed, the new community may function differently but it will result in the same process rates than observed in the original community^59^. Thus even if the active bacteria and fungi were less numerous following biostimulant addition in our study, possibly due to both redundancy of this heterotrophic function and the higher bacterial and fungal diversities we measured, the C mineralization was not impacted.

Nielsen et al.^56^ highlighted that the phylogenetic composition (identities) of the microbial communities often have stronger effects than total species richness and diversity on processes associated with C cycling such as decomposition or heterotrophic respiration. We demonstrated that, during the mineralization process, the biostimulant induced significant changes in the composition of active bacterial and fungal communities while, considering the cumulative C–CO_2_ emissions, the organic mineralization was unchanged. However, in the present study, the daily emissions of C–CO_2_ at the earlier stage were higher than at the latter stage. As highlighted above, changes in the microbial community without impacting the function could be explain by functional redundancy or the new community that might be functionally similar to the original. Along the incubation we observed bacterial and fungal successions that occurred simultaneously as reported by other authors^60–62^. These microbial successions are supported by the ecological concept of r/K-strategists^63^. They consist in a rapid growth rate at the earlier stages of the organic matter decomposition when the labile resource was abundant (i.e. copiotrophic/r-strategists), the daily emissions of C–CO_2_ being higher than at the latter stage in the present study. And, at the later stage, there is a stimulation of slow-growing microorganisms that have a greater ability to degrade recalcitrant organic matter and were thus able to survive and compete when resources were limited (i.e. oligotrophic/K-strategists)^64^.

With respect to the fungi in the bare soil, until 7 days of incubation, which corresponded in the present study to a pivotal time for the active microbial abundances, the abundance of active *Ascomycota* was reduced to the advantage of active *Zygomycota* (*Mortierellaceae)* and *Basidiomycoya*. In presence of BS, the decrease of the active *Ascomycota* abundances was less important while the activation of *Mortierellaceae* and *Basidiomycota* was more important. Interestingly, the *Ascomycota* are known to be more abundant in soils of moderate and lowly access carbon content (recalcitrant C) while *Basidiomycota* are stimulated in soils with high labile carbon content^24^; also, *Mortierellaceae* are known to be soil saprophytes exhibiting both copiotrophic and oligotrophic lifestyles^65,66^. Therefore, in the present study either at the phylum or family levels, the addition of biostimulant activated saprophyte microorganisms but any specific functional strategy toward the organic carbon mineralization [either oligotrophic (K-strategists) or copiotrophic (r-strategists)] could be demonstrated.

### Delayed positive effect of the biostimulant on the active microbial community

Whatever the addition of straw, the input of biostimulant to soil significantly increased the abundances of bacteria and fungi after 49 days of incubation. This highlighted a delayed positive effect of the biostimulant on soil active microorganisms. Our observation contradicts previous works focused on soil enzymatic activities as a proxy of microbial activity that showed rapid response of soil systems following biostimulant input. For example, higher soil enzyme activities (i.e. dehydrogenase activity, alkaline phosphatase and β-glucosidase) were observed already 24 h after the addition of biostimulants derived from wheat-condensed distiller solubles or from different sewage sludges, this positive effect starting to decline after 7 days^15,16^. The β-glucosidase is an enzyme used to assess the organic matter decomposition^67^ and as we showed the biostimulant did not increase the organic carbon mineralization along the incubation. So far, the present study is the first one to demonstrate such a delayed positive effect of a biostimulant on soil microorganisms. The biostimulant can induce changes in some soil physico-chemical characteristics such as the pH or the moisture that are known to be strong drivers of the soil microbial community^68–71^. In a previous study, focusing on another soil biostimulant, we showed that after 49 days of incubation the BS had a pH neutralizing effect that might have induced changes in the bacterial and fungal communities^6^. In the present study, the biostimulant had approximately one unit pH higher than the soil. We could therefore expect to again observe a pH neutralizing effect with the biostimulant tested. Laminarans which are expected to compose the biostimulant (e.g. peak of glucose after methanolysis) could argue for a pH neutralizing effect with the biostimulant tested as it has been reported that laminarans increased the incubation medium pH of grapevine cells suspension^72^. Although, soil pH monitoring along the incubation would have been needed to demonstrate such a neutralizing effect. Additionally, the high content of polysaccharides that the biostimulant contained may explain the delayed effect on soil active microorganisms, the release of glucose and galactose after the biotimulant methanolysis likely being from the laminarans and carrageenans contained in seaweeds, respectively. The carrageenans through their excellent gel forming properties are known to have specific slow release properties that are widely used in slow-acting pharmaceutical^73,74^. The carrageenans expected to be present in the biostimulant (e.g. peak of galactose after methanolysis) are also hydrophilic colloids and could therefore affect soil properties (physical, chemical and biological) by improving for example the moisture-holding capacity^19,74^. Indeed, moisture is known to strongly shape soil microorganisms by increasing their interaction with their environment, sustaining their abilities to obtain resources and disperse^71^. In addition the biostimulant that we used contains amino acids that can be used in protein synthesis or be directly absorbed by the microorganisms as an alternative source of nitrogen and carbon and increased the abundance of active microbial community^75,76^.

In the presence of the BS we showed that the abundance of active *Bacillaceae* increased up to a maximum at 49 days while without BS their abundance decreased with time. Several species belonging to the genus *Bacillus* are known to be plant growth promoting rhizobacteria (PGPR) which indirectly contributes to increase crop productivity by synthesizing plant hormones, fixing atmospheric nitrogen, solubilizing soil phosphorus, suppressing plant pathogens and insect pests (reviewed in^77^). During the last years, species from the genus *Bacillus* have attracted considerable attention for sustainable agriculture due to their various ways to promote plant growth and their great ability to maintain themselves in rhizospheric soil. These properties confer them advantages over other PGPR strains used in inoculant formulations. In the present study, bacteria from the genus *Bacillus* were not inoculated along with the biostimulant. The biostimulant activated some indigenous soil members of this taxon which has been reported to be more efficient than inoculation of exogenous *Bacillus* to increase plant stress tolerance^78^. Indeed, the environmental adaptation of the indigenous microorganisms should increase their ability to improve plant fitness compared to exogenous microorganisms^79^. Furthermore, the addition of either the biostimulant or the straw activated the *Cellviobrionaceae* that are known to be soil bacteria saprophytes with a high capacity to degrade various plant polysaccharides through production and excretion of specific enzymes (i.e. hemicellulose, cellulose, xylan, mannan, etc.)^80^ suggesting again a generic effect of the biostimulant on saprophytes and not a specific one. This result is consistent with our previous findings on the effect of another soil biostimulant in which either oligotrophic or copiotrophic soil bacteria and soil fungi were stimulated^6^. In the present study, the biostimulant that we tested is intended to be applied on crop residues, between two crops, to increase their mineralization and the further release of nutrients. Hence the next crop will take benefit from the delayed changes in microbial communities induced by the biostimulant and involving growth promoting bacteria and soil saprophytes.

### Effects of the BS on soil microorganisms relative to plant legacy effects

Plants shape the composition of soil microorganisms through the release of root exudates^81^ that correspond to the deposition of fresh organic carbon derived from products of photosynthesis^82^ and through the modification of physico-chemical properties of the soil^83^. The root-derived organic inputs are discussed as the main drivers of soil communities^84^ and the changes they induced are still detectable in the absence of the organism, this persistent effect of ecological interaction is known as legacy effect^85^. In this study contrasted legacy effects of *A. thaliana* and *T. aestivum* were measured and were compared with the delayed effect of the BS that we observed at the long term (49 days). We first showed that the soil active fungal were differentially impacted by the plant legacy effect and by the biostimulant. At the end of the planted soil phase, the organic carbon content was significantly higher in soils that grew both plants than in the bare soil (data not shown) and could be attributed to the root deposition of fresh organic carbon^82^. While the input of organic carbon in soil by the biostimulant was negligible (i.e. 37.85 µg organic carbon) the effects of the BS and those induced by the two plants on soil microorganisms were of similar extent. As organic carbon content was higher in the planted soils we could have expect higher abundances of active bacteria and fungi. Indeed, the root release of organic carbon in soils directly influence soil microorganisms by providing them a new source of labile energy^86^. Otherwise, the plant could indirectly shape microbial community by modifying for example the soil pH, the nutrient availability or the soil moisture^83^. Regarding the biostimulant effect at the long term (49 days), it could be attributed to its chemical composition, especially its carrageenans content, which could indirectly influence soil microbial community by inducing changes in the physico-chemical soil properties, as discussed above. Moreover, at the medium-term of 49 days, the biostimulant significantly increased the richness and diversity of fungi compared to plant legacy. This is of interest as soil biodiversity conservation have an important role to ensure the sustainability and the recovering of a function (i.e. resilience) after environmental disturbances^87^.

This study demonstrated that the biostimulant input to soil did not inoculate specific microorganisms but immediately reduced the amount of active microorganisms. However, at the medium-term of 49 days the BS had a delayed beneficial effect on active native soil microorganisms and activated saprophytes bacteria and fungi and bacteria known to promote the plant growth. However, the changes that we observed in active microbial abundances, richnesses and diversities were not associated to an increase in the mineralization rate of the organic matter in our controlled conditions and might be explained by low PO_4_^3−^ content in soil. Finally, we showed that after 49 days, the biostimulant effect could be similar or even higher than those of plants. So far, the present study is the first one that demonstrates such a delayed positive effect of a biostimulant on active soil microorganisms which should be beneficial for accompanying agroecological practices.

## Supplementary information

Supplementary Information.
