# Supplementary material for: Responses of active soil microorganisms facing to a soil biostimulant input compared to plant legacy effects
Source: Sci Rep. 2020 Aug 13;10:13727. doi: 10.1038/s41598-020-70695-7 (PMC7426422; doi:10.1038/s41598-020-70695-7)
Supplement: Supplementary file 1 — Supplementary Information. [file 41598_2020_70695_MOESM1_ESM.pdf]

Responses of active soil microorganisms facing to a soil biostimulant input compared to plant legacy effects

Eve Hellequin [1,*], Cécile Monard [1], Marion Chorin[1], Nathalie Le bris[1], Virginie Daburon[1], Olivier Klarzynski [2], Françoise Binet [1]

Eve Hellequin [1,*], Cécile Monard [1], Marion Chorin[1], Nathalie Le bris[1], Virginie Daburon[1], Olivier Klarzynski [2], Françoise Binet [1*]

1. University of Rennes, CNRS, ECOBIO [(Ecosystèmes, biodiversité, évolution)] - UMR 6553, F-35000 Rennes, France.

2. BIO3G Company, 7 rue du Bourg-Neuf 22230 Merdrignac, France.

* Corresponding authors:

-Eve Hellequin (current address: University of Sorbonne, CNRS, EPHE, PSL, UMR METIS, F-75005 Paris, France. (eve.hellequin@upmc.fr)

-francoise.binet@univ-rennes1.fr

Table S1: Main biomolecules detected in the biostimulant. Contents are expressed on 105 °C dried matter (means and standard deviations, n=3). dw: dry weight. DL: detection limit.

	**Contents (g 100g dw^{-1})**
**Total Polysaccharides**	**40.2 ± 2.5**
Glucose	20.7 ± 1.1
Galactose	18.8 ± 1.3
Mannose	0.7 ± 0.1
**Total Monosaccharides**	**3.6 ± 0.1**
Galactose	3.6 ± 0.1
Glucose	0 < (DL)
Mannose	0 < (DL)
**Total Amino acids**	**9.3**
**Alanine**	**0.81**
Arginine	0.34
**Aspartic acid**	**0.94**
Cystine	0.05
Glutamic acid	0.74
Glycine	0.39
Histidine	0.60
Isoleucine	0.03
**Lysine**	**0.87**
**Leucine**	**1.24**
Methionine	0.13
Phenylalanine	0.72
Proline	0.32
Serine	0.44
Threonine	0.43
Tryptophan	0.16

Tyrosine	0.24
**Valine**	**0.84**

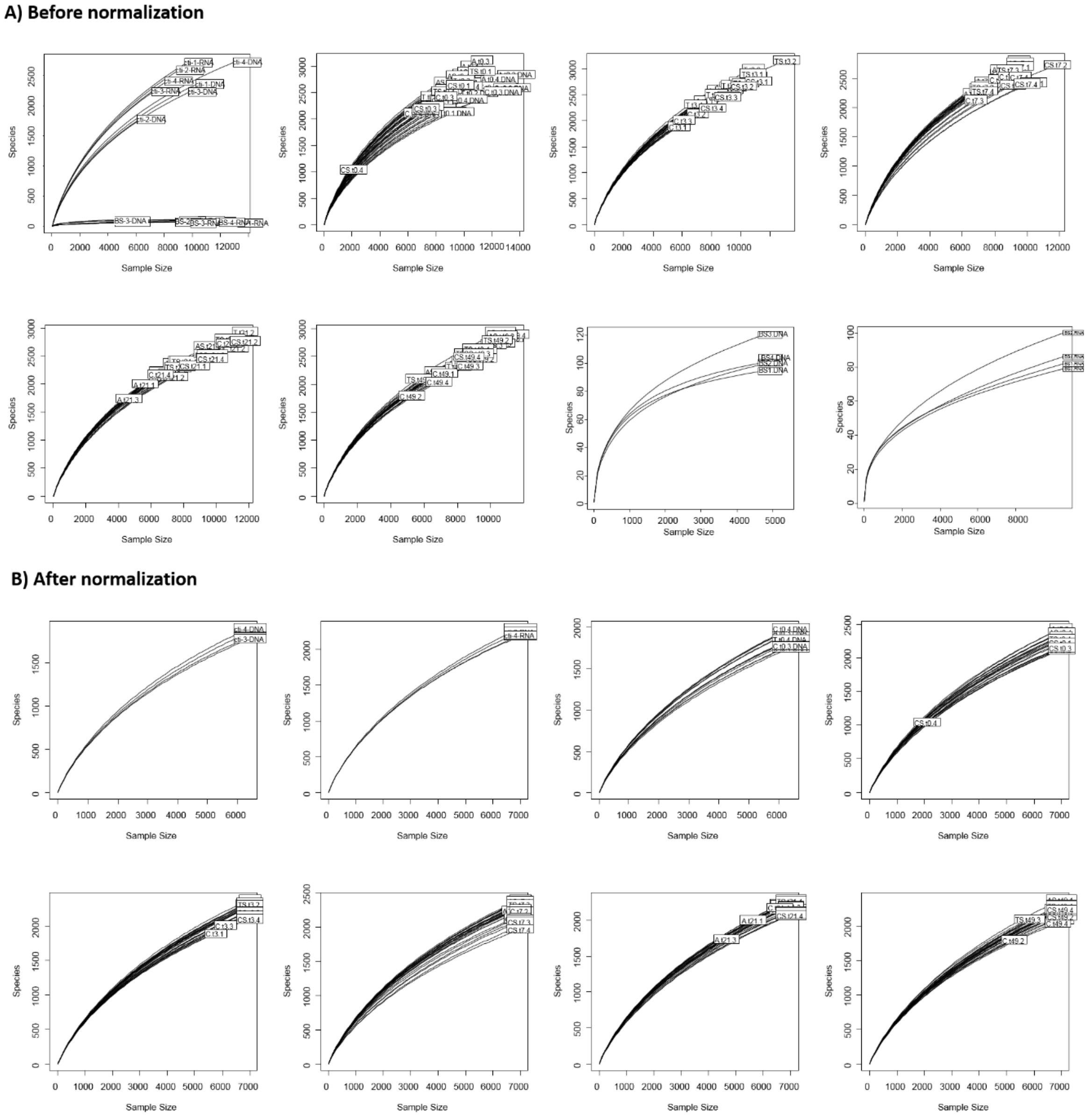

Figure S1. Rarefaction curves of total and active bacteria (from DNA and cDNA respectively) at the different times during the soil incubation and before (A) and after normalization (B). C=bare soil, CS: bare soil with straw, A: *A. thaliana* soil, AS: *A. thaliana* soil with straw, T: *T. aestivum* soil, TS: *T. aestivum* soil with straw, BS: raw biostimulant. The different sampling times were t = i, 0, 3, 7, 21 and 49 days, i: initial soil before soil plant-growing**.**

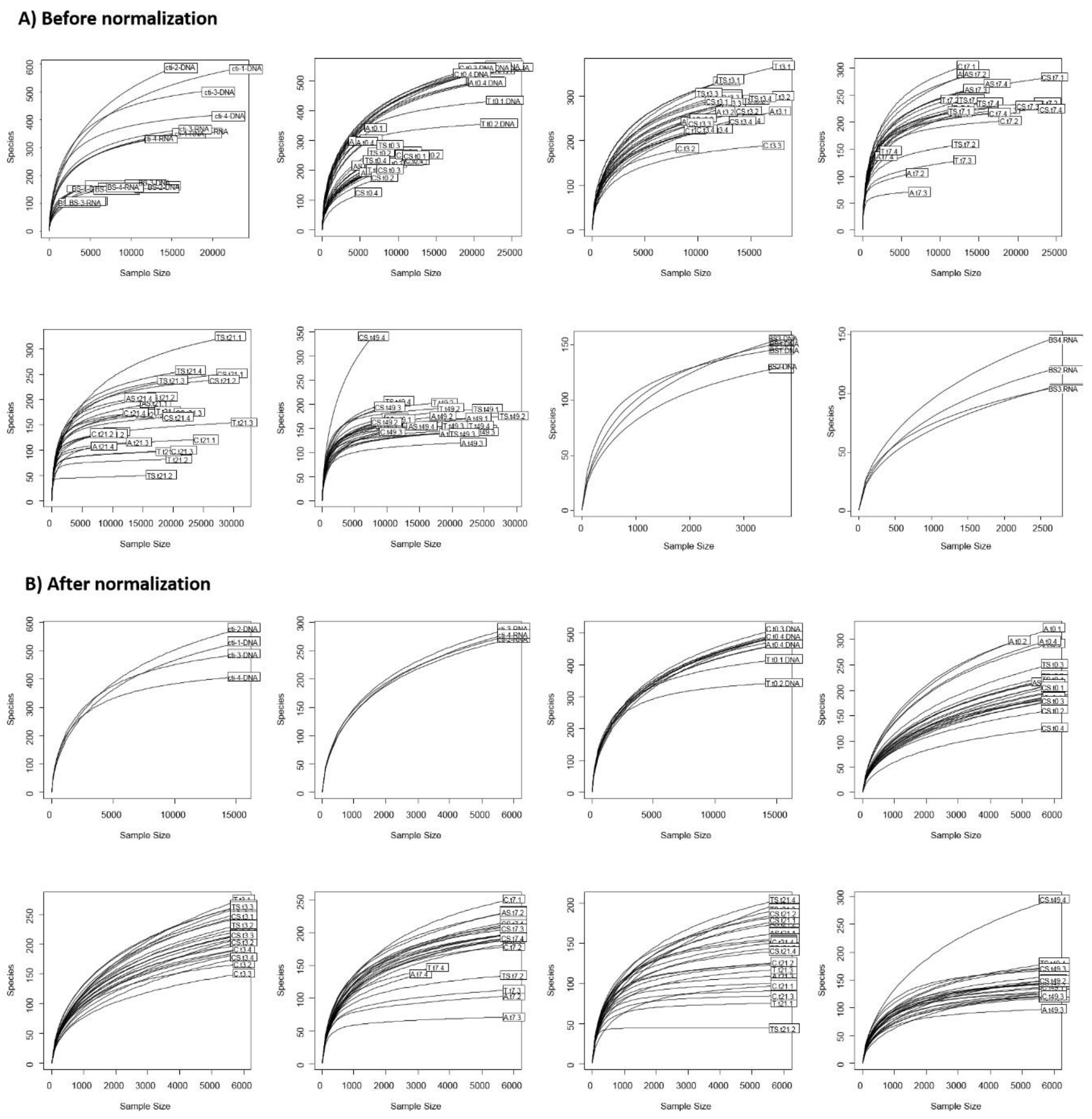

Figure S2. Rarefaction curves of total and active fungi (from DNA and cDNA respectively) at the different times during the soil incubation and before (A) and after normalization (B). C: bare soil, CS: bare soil with straw, A: *A. thaliana* soil, AS: *A. thaliana* soil with straw, T: *T. aestivum* soil, TS: *T. aestivum* soil with straw, BS: raw biostimulant. The different sampling times were t = i, 0, 3, 7, 21 and 49 days, i: initial soil before soil plant-growing**.**

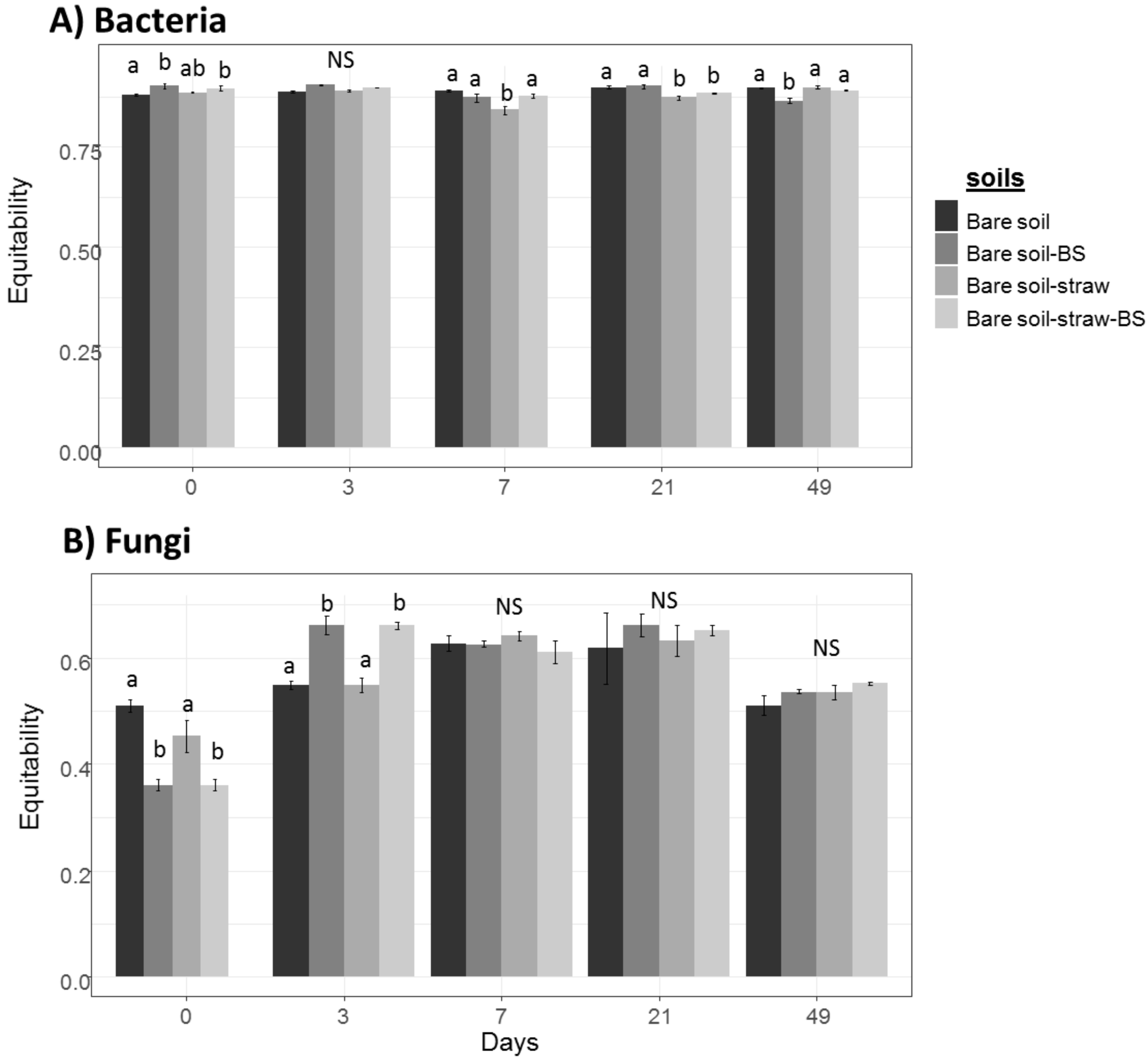

Figure S3: Equitability index of active bacterial and fungal communities in the bare soil with and without straw and/or BS. The statistical analyses were performed between soils at each sampling date. The error bars indicate the standard errors and the different letters indicate significant differences according to ANOVA and Tukey's tests, NS: non-significant.

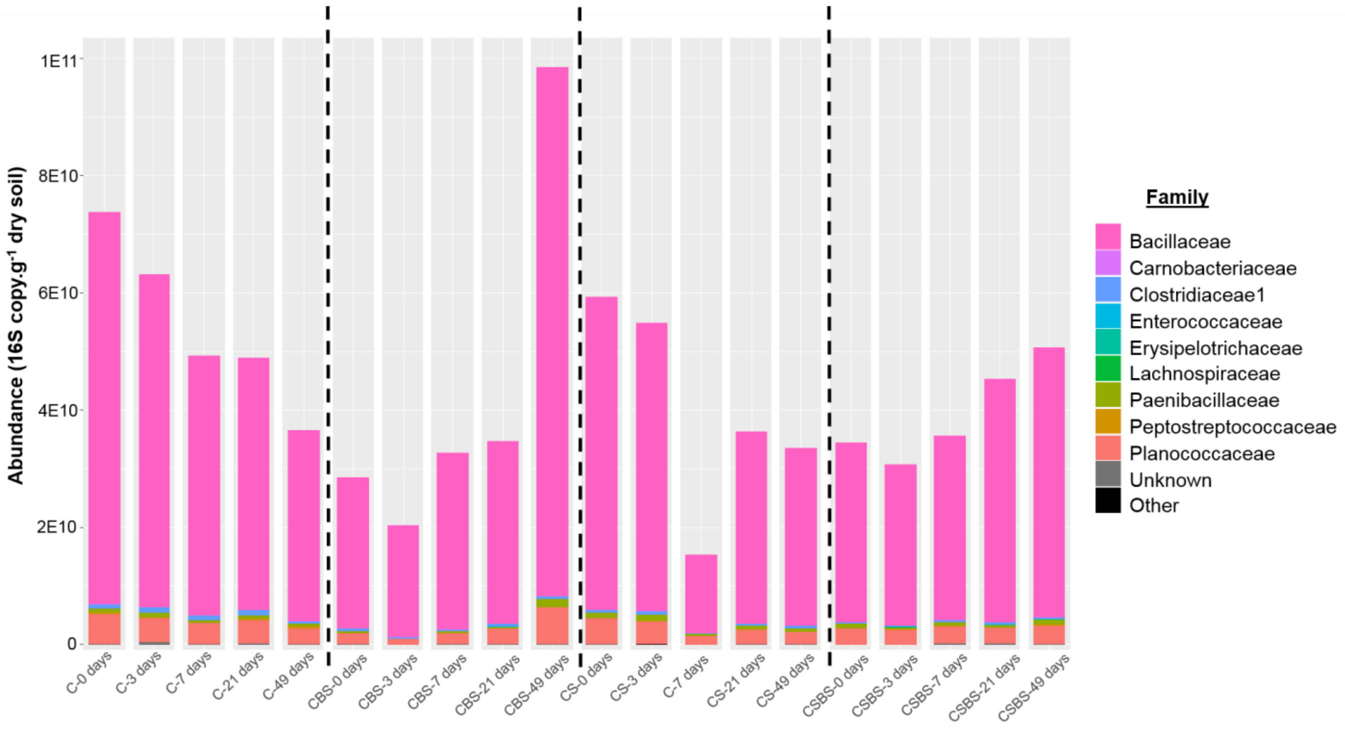

Figure S4: Composition of the *Firmicutes* phylum: abundances of the families that composed it at the beginning of incubation and after 3, 7, 21 and 49 days of incubation in the different soils. C: bare soil, CS: bare soil with straw, CBS: bare soil with raw biostimulant, CSBS: with straw and biostimulant.

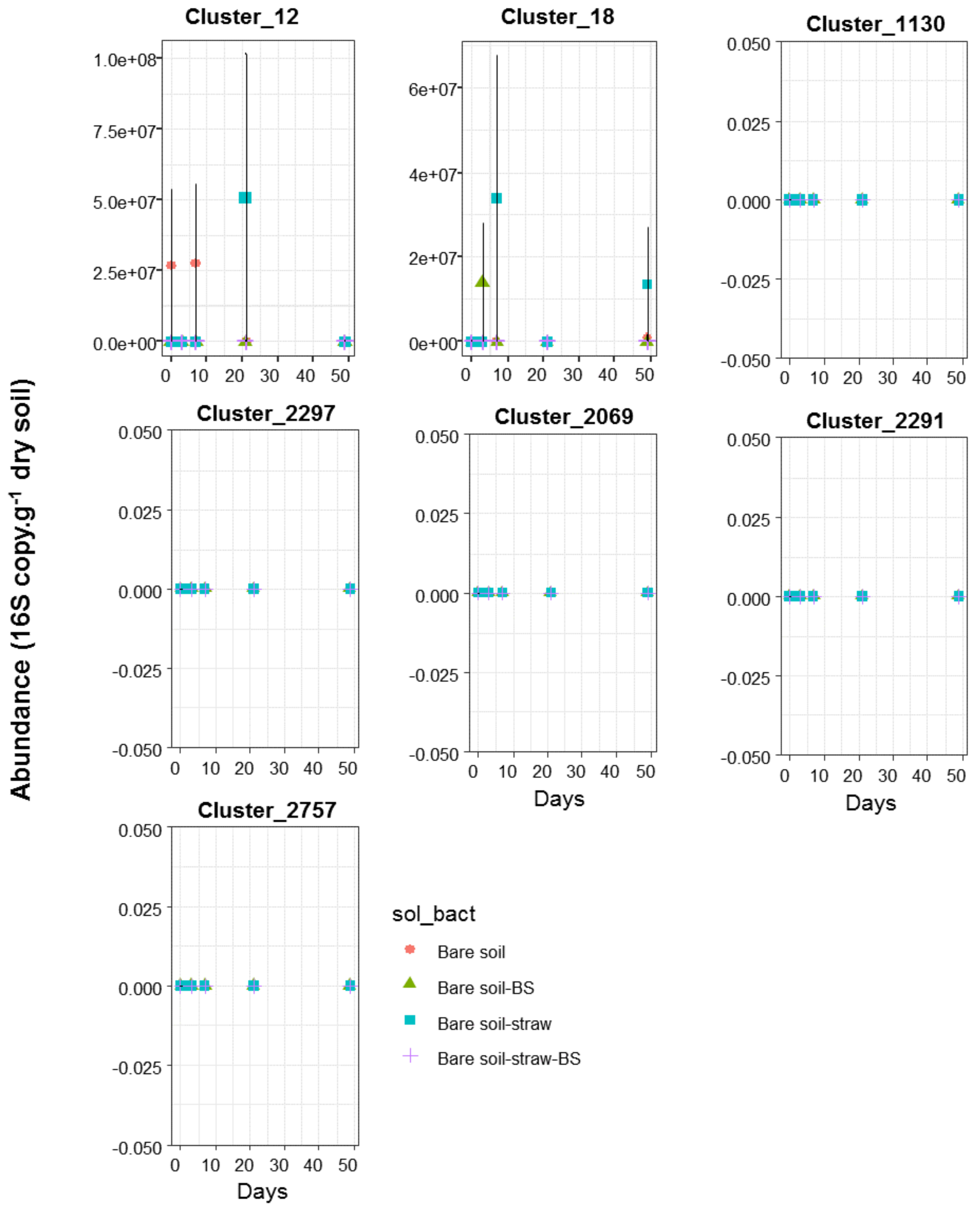

Figure S5: Abundance dynamics of some OTUs, identified as highly present and/or active in the raw biostimulant, in the different soil treatments at the beginning of incubation and after 3, 7, 21 and 49 days of incubation The error bars indicate standard errors (n=4).

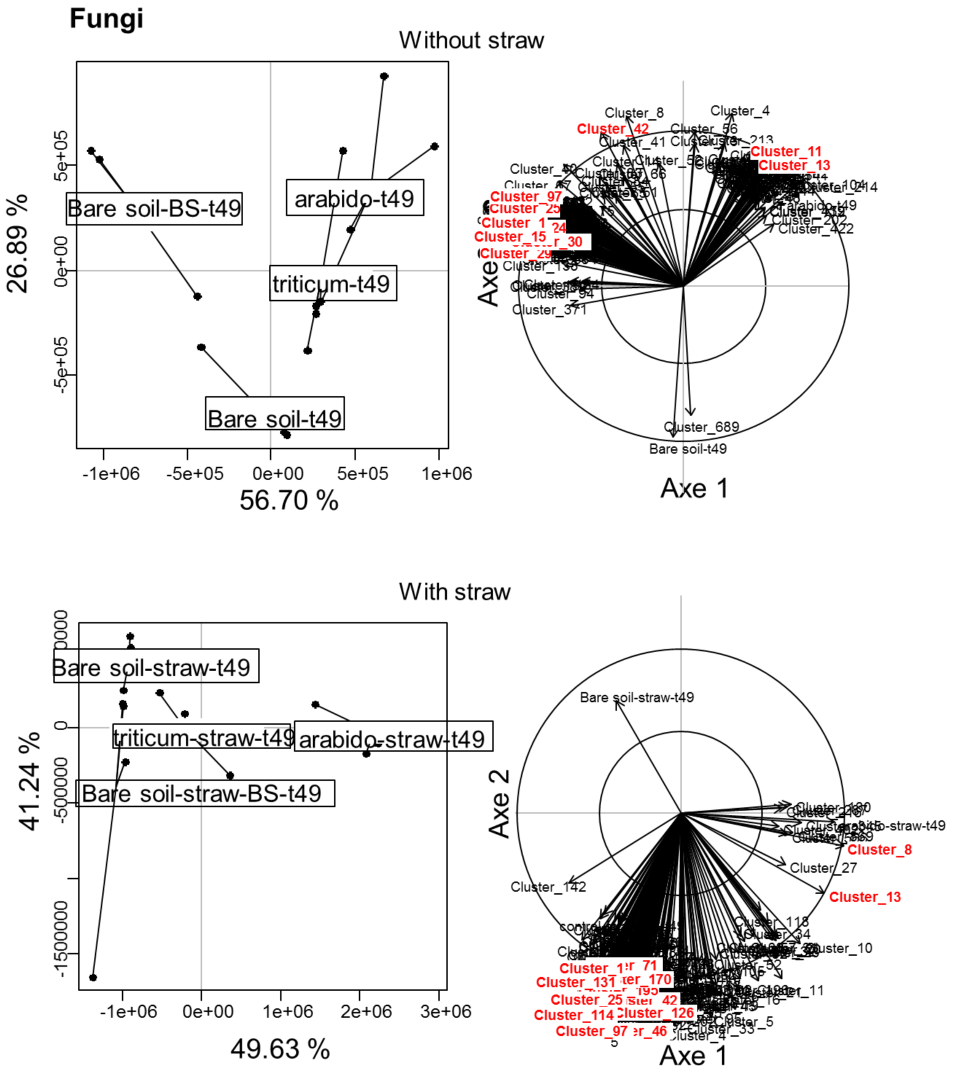

Figure S6: Powered partial least square discriminant analysis (PPLS-DA) describing the fungal community structures at the OTU level. The analysis was generated at day 49 in the soils without and with straw. In the correlation circles, the OTUs in red correspond to the most abundant OTUs that contributed significantly to the differences observed between soils according to ANOVAs.
